# Efficacy of Switching to Adalimumab for Maintenance of Remission Following Induction Therapy with Tacrolimus in Patients with Ulcerative Colitis

**DOI:** 10.3390/jcm12206699

**Published:** 2023-10-23

**Authors:** Keijiro Numa, Kazuki Kakimoto, Yasuyoshi Tanaka, Noboru Mizuta, Naohiko Kinoshita, Kei Nakazawa, Ryoji Koshiba, Yuki Hirata, Kazuhiro Ota, Takako Miyazaki, Shiro Nakamura, Kazuhide Higuchi, Hiroki Nishikawa

**Affiliations:** 2nd Department of Internal Medicine, Osaka Medical and Pharmaceutical University, 2-7 Daigakumachi, Takatsuki City 569-8686, Japan

**Keywords:** adalimumab, tacrolimus, ulcerative colitis

## Abstract

Background: Tacrolimus (TAC) effectively induces remission in refractory ulcerative colitis (UC). However, TAC therapy usually lasts for 3 months. Although azathioprine (AZA) is often used in maintenance therapy, the relapse rate remains high. Herein, we evaluated the efficacy of adalimumab (ADA) for remission maintenance in patients with UC after induction therapy with TAC. Methods: We prospectively enrolled patients with moderate-to-severe UC who achieved clinical remission after 3 months of TAC therapy with endoscopic non-mucosal healing (Cohort A). After TAC discontinuation, the remission maintenance rate up to 1 year after starting ADA therapy was examined. We retrospectively enrolled patients with UC treated with TAC (Cohort B). Among patients in clinical remission after TAC treatment for 3 months, those who received AZA as remission maintenance therapy after TAC discontinuation constituted the AZA group. Patients in Cohort A who received ADA and AZA as remission maintenance therapy after TAC discontinuation constituted the ADA + AZA group. We compared the remission maintenance rates in the AZA and ADA + AZA groups for up to 5 years after TAC discontinuation. Results: In Cohort A, of the 46 patients with UC treated with TAC, 17 were eligible for analysis after receiving ADA as remission maintenance therapy. A notable 88.2% (15/17) were still in remission 1 year after starting ADA. The ADA + AZA group (*n* = 16) exhibited a significantly higher relapse-free rate than the AZA group (*n* = 26) (*p* < 0.05; log-rank test). Conclusion: switching to ADA for remission maintenance in patients with refractory UC who achieved clinical remission with TAC is clinically useful.

## 1. Introduction

Ulcerative colitis (UC) is characterized by inflammation of the colon, involving cycles of remissions and relapses [1]. Tacrolimus (TAC), a calcineurin inhibitor, has a high remission induction rate in patients with steroid-dependent, resistant, moderate-to-severe UC [2,3,4]. For achieving remission, TAC therapy generally lasts for 3 months; however, most patients experience relapse after discontinuing TAC [5,6]. Remission maintenance is crucial in ulcerative colitis because prolonged related chronic inflammation adversely impacts quality of life and increases the risk of developing colorectal cancer [7,8,9]. The usefulness of azathioprine (AZA), an immunomodulatory agent, as remission maintenance therapy after TAC discontinuation has been reported [10,11], and AZA is commonly used in clinical practice; however, the relapse rate remains high despite AZA therapy [12]. A high relapse rate has been reported in patients in whom endoscopic mucosal healing had not been achieved by the time of TAC discontinuation [13]. However, TAC therapy is indicated for refractory UC with high disease activity, with few patients achieving complete mucosal healing even 3 months after the initiation of therapy. Therefore, it is necessary to introduce maintenance therapy to prevent UC relapse, especially in patients who fail to achieve mucosal healing despite TAC treatment. Pellet et al. retrospectively evaluated the efficacy of vedolizumab (VDZ) as maintenance remission therapy in patients with UC who responded to and reported favorable outcomes after calcineurin inhibitors (cyclosporine or tacrolimus) were used as induction remission therapy [14]. To be considered a suitable agent for remission maintenance therapy after TAC treatment, a drug must demonstrate long-term efficacy and a strong safety profile. The effectiveness of adalimumab (ADA), an anti-TNFα drug, in sustaining long-term remission in UC has been demonstrated [15,16]. Moreover, there have been numerous reports on the presence of a few anti-drug antibodies and the high safety of ADA [17,18,19,20]. Therefore, ADA is considered a promising therapeutic candidate for maintaining remission after TAC therapy. However, the potential benefits of switching to ADA for remission maintenance after TAC treatment have not been explored. Herein, the usefulness of a new treatment strategy (switching to ADA) for maintaining remission in patients with refractory UC, who achieved clinical remission with TAC treatment, is investigated.

## 2. Materials and Methods

### 2.1. Statement of Ethics

This study was performed in accordance with the principles outlined in the Declaration of Helsinki. The study protocol was approved by the Ethics Committee of Osaka Medical and Pharmaceutical University (no. 2155-3). Written informed consent was obtained from all patients included in the study.

### 2.2. Study Design and Patients

Cohort A (switching to ADA after remission induction therapy with TAC)

Among patients with moderate-to-severe UC who received TAC as remission induction therapy at Osaka Medical and Pharmaceutical University Hospital between October 2014 and December 2016, those who achieved clinical remission 3 months after the initiation of TAC and had not yet achieved endoscopic mucosal healing were prospectively enrolled. The main exclusion criteria were as follows: (1) patients with a history of treatment with anti-TNFα antibody agents; (2) patients with diseases listed in the package insert as contraindications or warnings against ADA administration, such as serious infections, demyelinating diseases, or malignant tumors; and (3) patients who did not provide consent for study participation. Patients who met the inclusion criteria and were enrolled in the study were started on ADA for remission maintenance after TAC was discontinued through dose tapering 3 months after treatment initiation. Patients who had already received AZA at the initiation of ADA were continued on AZA, whereas those who had not received AZA were simultaneously initiated on AZA. Patients with a history of adverse reactions to AZA were not administered the drug. The primary endpoint was the sustained remission of UC 1 year after initiation of ADA; secondary endpoints were the surgical procedure rate and incidence of side effects.

Cohort B (comparison of AZA and ADA + AZA as therapies for maintaining remission after TAC discontinuation)

Among patients with moderate-to-severe UC treated with TAC at Osaka Medical and Pharmaceutical University Hospital between December 2007 and December 2016, those who achieved clinical remission 3 months after receiving TAC and were treated with AZA as maintenance therapy after the discontinuation of TAC were enrolled retrospectively. The patients who received AZA alone after TAC discontinuation constituted the AZA group. Patients in Cohort A who received ADA and AZA combination maintenance therapy after TAC discontinuation constituted the ADA + AZA group. The number of patients that achieved and maintained remission for up to 5 years after the discontinuation of TAC in both groups was compared. In addition, risk factors related to clinical relapse after the discontinuation of TAC were identified and analyzed.

### 2.3. Drug Administration

In all of the patients in Cohorts A and B, TAC was orally administered twice daily at an initial dose of 0.1 mg/kg/day. Serum trough concentrations were maintained at 10–15 ng/mL for 2 weeks, then 5–10 ng/mL for 3 months. As appropriate, serum trough concentrations were measured and TAC doses were adjusted. Three months after the initiation of TAC, the dose was tapered and then discontinued. In Cohort A, all patients initially received ADA at a dose of 160 mg, followed by 80 mg after 2 weeks, subcutaneously, and subsequently 40 mg subcutaneously every 2 weeks; the initial dose of AZA was 1 mg/kg but was increased at the discretion of the treating physician.

### 2.4. Definitions

The Lichtiger index was used to assess clinical disease activity [21,22]. Clinical remission was defined as a Lichtiger index of 3 points or less. The ulcerative colitis endoscopic index of severity (UCEIS) was used to assess endoscopic disease activity, with endoscopic mucosal healing defined as a UCEIS score of 0 points [23,24,25]. Clinical relapse was defined as the need for remission-inducing therapy, treatment escalation, hospitalization, or colectomy.

### 2.5. Statistical Analysis

Quantitative data were summarized using the median and interquartile range (IQR), while categorical variables were presented using frequencies and percentages. We used Fisher’s exact test or Pearson’s chi-square test for categorical variables and the Mann–Whitney U test for continuous variables to compare demographic variables. The rate of maintaining clinical remission was analyzed using Kaplan–Meier analysis, and differences between groups were evaluated using the log-rank test. The risk factors for relapse after TAC treatment were calculated as odds ratios (ORs) with 95% confidence intervals (CIs) using logistic regression analysis. Statistical significance was set at *p* < 0.05 (two-tailed test). All statistical analyses were performed using JMP^®^, Version 15.2.1, SAS In-stitute Inc., Cary, NC, USA, 1989–2021.

## 3. Results

### 3.1. Cohort A

#### 3.1.1. Patient Characteristics

**Figure 1:**
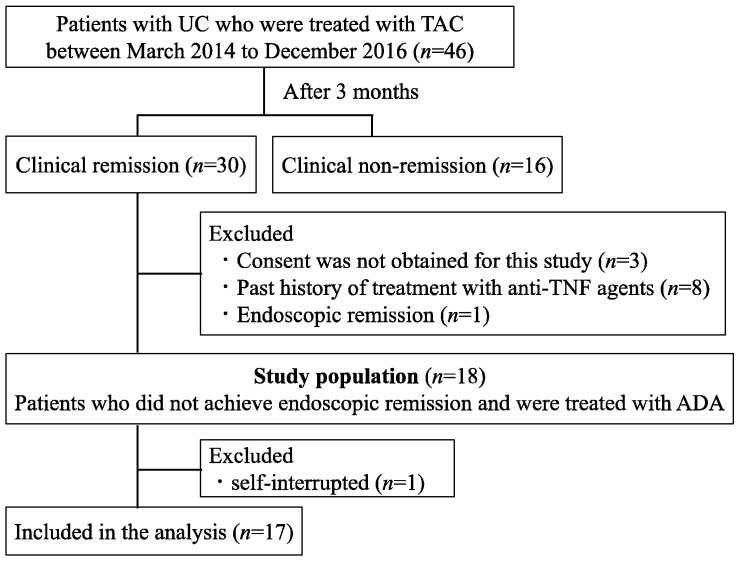
Patient flowchart for Cohort A. Forty-six patients received tacrolimus treatment from October 2014 to December 2016. Of these, 17 were in clinical remission but not endoscopic remission after 3 months and could be switched to ADA as therapy to maintain remission.

Of the 46 patients with refractory UC treated with TAC during the study period, 30 achieved clinical remission after 3 months. Among these, 18 patients were enrolled, excluding 8 patients who had previously used anti-TNFα antibody preparations, 1 patient who had achieved endoscopic mucosal healing as confirmed via colonoscopy findings 3 months after TAC administration, and 3 patients who did not provide consent to participate. ADA was administered to maintain remission. Finally, 17 patients were included in the analysis, after excluding 1 patient who discontinued ADA after initiation at his discretion (Figure 1).

The clinical characteristics of the patients are presented in Table 1. The median age was 48 years, with 58.8% of the patients being male, and the median (IQR) UC duration was 5 years (2–15 years). Among all patients with UC included in the study, 70.6% had pancolitis. At the initiation of TAC therapy, 15 patients (88.2%) were being treated with 5-ASA, 5 (29.4%) were being treated with AZA, and 6 (35.5%) were being treated with prednisolone (PSL). The median (IQR) C-reactive protein (CRP) level at the initiation of TAC was 1.18 (0.14–10.66), and the Lichtiger CAI was 13 (10–14). The median (IQR) CRP and Lichtiger CAI at the start of ADA therapy were 0.07 (0.03–0.14) and 1 (1–3), respectively. Fifteen patients were treated with ADA and AZA for maintaining remission, except for two patients who exhibited intolerance to AZA and could not receive the combination regimen.

#### 3.1.2. Maintenance of Clinical Remission up to 1 Year after ADA

**Figure 2:**
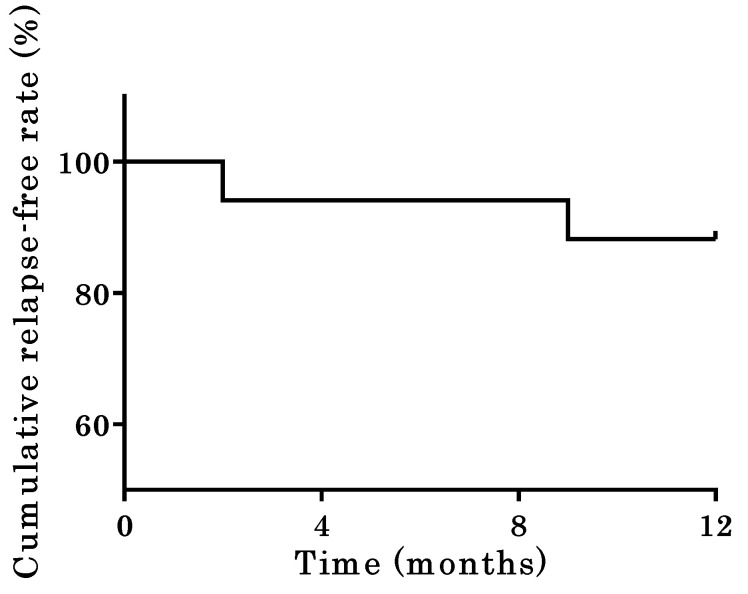
Relapse-free survival up to 1 year after starting adalimumab as therapy for maintaining remission after the discontinuation of tacrolimus therapy.

Figure 2 shows the Kaplan–Meier curve for relapse-free survival up to 1 year after starting maintenance therapy with ADA. Two patients (11.8%) experienced a clinical relapse of UC during the first year after the introduction of ADA, while fifteen (88.2%) remained in remission. Of the two patients who relapsed, one responded positively to 5-ASA enema and continued ADA treatment. The other patient switched treatment to infliximab (IFX). None of the patients underwent surgery, and no adverse effects related to ADA administration were reported.

### 3.2. Cohort B

#### 3.2.1. Patient Characteristics

**Figure 3:**
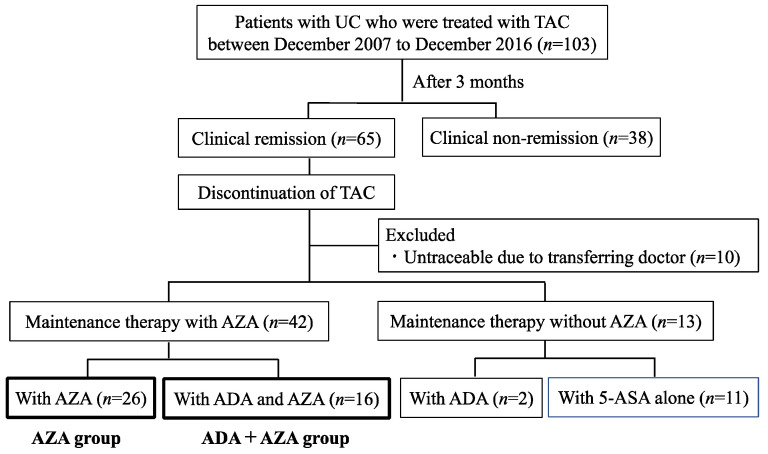
Patient flowchart for Cohort B. A total of 103 patients received tacrolimus treatment from December 2007 to December 2016. Of these, 26 (AZA group) achieved clinical remission after 3 months and maintained remission with AZA alone, while 16 (ADA + AZA group) maintained remission with ADA plus AZA.

Throughout the study period, TAC was administered to 103 patients with refractory UC, and after 3 months of treatment, clinical remission was achieved in 65 patients. After the discontinuation of TAC, 26 patients (AZA group) received maintenance therapy with AZA alone, and 16 patients (ADA + AZA group) received ADA in combination with AZA (Figure 3). Table 2 shows a comparison of patient characteristics at TAC initiation between the AZA and ADA + AZA groups. No significant differences were observed in sex, age, disease duration, or disease type between the two groups. The Lichtiger indexes at the initiation of TAC therapy exhibited no significant difference between the AZA and ADA + AZA groups (median [IQR] 13 [11–15.5] vs. 13 [10–14], *p* = 0.43). Furthermore, no significant differences were observed in the blood and biochemical findings between the two groups.

#### 3.2.2. Comparison of Clinical Remission 5 Years after Discontinuation of TAC between the AZA and ADA + AZA Groups

**Figure 4:**
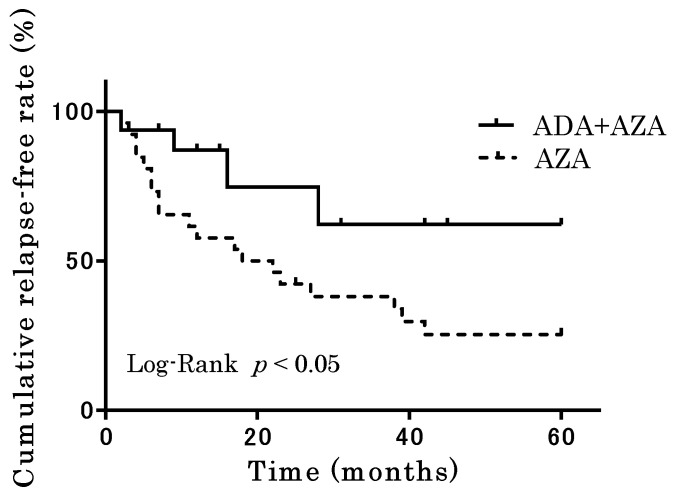
Comparison of the AZA and ADA + AZA groups in terms of relapse-free survival up to 5 years after the discontinuation of tacrolimus.

Clinical relapse was observed in 19 (73.1%) patients in the AZA group and 4 (25%) patients in the ADA + AZA group during the observation period. Figure 4 shows relapse-free survival in the AZA and ADA + AZA groups 5 years after TAC discontinuation. The overall relapse-free rate was significantly higher in the ADA + AZA group than in the AZA group (*p* < 0.05; log-rank test). In the AZA group, 13 patients received remission-inducing therapy, 2 patients were hospitalized, 1 patient received treatment escalation, and 3 patients received colectomy as treatment after relapse. In the ADA + AZA group, three patients received remission-inducing therapy, and one patient received colectomy as treatment.

#### 3.2.3. Comparison of Clinical Characteristics in the Remission and Relapse Groups

To identify the risk factors for relapse after discontinuing TAC, we categorized 42 patients enrolled in Cohort B into two groups: those who maintained remission and those who relapsed. Their clinical characteristics were compared until 5 years after the discontinuation of TAC. The remission and relapse groups comprised 15 and 27 patients, respectively, with no significant differences in clinical characteristics between the two groups. Notably, no significant differences were evident in the Lichtiger index or blood biochemical parameters at TAC initiation. Significantly more patients in the remission group received ADA + AZA for maintaining remission after TAC discontinuation (*p* = 0.03) (Table 3). Logistic regression analysis, used to examine factors predicting the maintenance of clinical remission after TAC discontinuation, revealed a significant association between ADA + AZA administration and the maintenance of clinical remission (OR = 4.3 [95% CI: 1.1–16.4], *p* = 0.03) (Table 4).

## 4. Discussion

In Cohort A, we prospectively evaluated the usefulness of ADA as therapy for maintaining remission after discontinuing TAC in patients with refractory UC who achieved clinical remission with TAC treatment but had not yet achieved endoscopic mucosal healing. The results indicated that switching from TAC to ADA was highly effective in maintaining remission. In Cohort B, the ADA + AZA group exhibited a significantly higher relapse-free rate than the control group, highlighting the usefulness of an ADA-based treatment strategy in maintaining remission after TAC discontinuation.

Although TAC is a highly effective remission-inducing agent for refractory UC with high disease activity [26,27,28], the high rate of UC relapse after discontinuation is concerning. TAC is specifically indicated for the treatment of severe refractory UC, which is characterized by a tendency to recur. Additionally, TAC is typically administered for 3 months, with few cases showing mucosal healing at the time of treatment discontinuation. In particular, many patients relapse early after discontinuing TAC, suggesting that mucosal healing might not have occurred at the time of discontinuation [13]. Therefore, particularly for patients who have not achieved mucosal healing at the time of TAC discontinuation, conventional AZA therapy used to maintain remission is insufficient. Thus, there is a clinical need for additional treatment or strategies. We believe that this study is of great significance because it proposes a new treatment strategy that can potentially fulfil the abovementioned clinical need.

In addition to ADA, alternative anti-TNFα antibody therapies indicated for the treatment of UC include IFX and GLM. ADA has been reported to be less effective in inducing remission among these therapies, but does have a certain level of efficacy in maintaining remission [15]. Moreover, ADA is a fully sequence-derived human antibody, indicating a low incidence of anti-drug antibody generation and a high long-term safety profile. The reason for using ADA in this study stems from our focus on patients with UC who achieved clinical remission through TAC treatment, to prioritize long-term safety and efficacy during the maintenance of remission. Concerns of overtreatment may arise regarding the administration of ADA to patients with UC already in clinical remission. However, in this study, ADA administration was limited to patients with UC manifesting residual disease activity, as confirmed endoscopically, and those with a high likelihood of UC relapse. For such cases, additional treatment may be of clinical importance in preventing early UC relapse. Some clinicians advocate initiating anti-TNFα antibody therapy as a dual strategy to induce and sustain remission instead of administering TAC. In hospitalized patients with acute severe UC, determining the most effective therapy out of the anti-TNFα antibody therapies, IFX, or the calcineurin inhibitors, cyclosporine or tacrolimus, to reduce the risk of colectomy is critical (AGA Guidelines). Although IFX is the most potent remission-inducing agent among biological therapies, it is still reported to have a high incidence of primary inefficacy in UC with high disease activity [29]. Notably, high inflammation in intestinal tissues increases the clearance of anti-TNFα antibody agents, resulting in lower drug concentrations [30]. In contrast, TAC has been shown to induce remission in patients with UC with high disease activity [2,3,4,26] and is an important therapeutic agent in specialized IBD centers owing to its capacity for individualized serum trough concentrations. Therefore, a reasonable treatment strategy entails the induction of remission with TAC followed by anti-TNFα antibody therapy for maintaining remission in hospitalized patients with UC related to high disease activity.

Pellet et al. retrospectively evaluated the efficacy of a calcineurin inhibitor (cyclosporine or tacrolimus) for inducing remission and vedolizumab (VDZ) as additional therapy for maintaining remission in 39 patients with steroid-resistant UC [14]. In the aforementioned study, most patients initially responded positively to calcineurin inhibitors, and VDZ was only administered if they responded adequately to induction therapy. The results showed that more than two thirds of patients did not need a colectomy. To the best of our knowledge, the current study is the first to report the administration of biological therapies for maintaining remission in patients with UC who responded to TAC therapy. VDZ is a monoclonal antibody against α4β7 integrin that inhibits lymphocyte infiltration into the gut by blocking the adhesion of α4β7 integrin on lymphocytes to MAdCAM-1 expressed in the gastrointestinal mucosa [31]. The gut-specificity of the drug makes it safe and easy for use as a long-term remission maintenance agent after the completion of TAC treatment. Conversely, the present study reports for the first time the administration of ADA in remission maintenance after TAC treatment. Ustekinumab (UST) may be a future alternative to ADA and VDZ. UST is a monoclonal antibody against p40, a subunit that is common to interleukin (IL)-12 and IL-23. UST suppresses chronic inflammation by inhibiting the expression of IL-12, which induces the T helper 1 cell response, and IL-23, which is involved in T helper 17 cell differentiation [32,33]. The use of UST for remission maintenance therapy after TAC treatment may be relatively easy owing to its relatively high safety profile and long-term remission maintenance effect.

Fecal microbiota transplant (FMT), a microbiome-based intervention that has attracted attention in recent years, may be effective as a remission maintenance therapy aside from biologics. FMT involves the transplantation of feces from a healthy donor to introduce beneficial microorganisms to the receiver. However, pre-cleaning of the resident gut microbiota with antibiotics and multiple doses may be required to sustain the desired impact [34,35]. Probiotics are live microbial supplements that may improve the host’s immune response by enhancing intestinal barrier function and improving the local immune response [36]. The administration of probiotics and antibiotics improves perioperative outcomes in UC [37,38,39]. However, the efficacy of probiotics in maintaining remission is unclear. The combined use of probiotics and 5-ASA may slightly improve remission maintenance compared to the sole use of 5-ASA [40]. In the present study, no difference was observed between the impact of probiotics in the remission and relapse groups. However, limited data were obtained from a small number of participants, and the evidence is unreliable. Future clinical trials should be large in scale based on a large number of cases.

This study has several limitations. First, the study design should have involved a prospective comparison of therapeutic efficacy in maintaining remission by categorizing patients into two groups: one receiving ADA and the other not, after the discontinuation of TAC. The reason for choosing a single-arm study design with ADA stems from the single-center nature of the research and limited availability of patients with UC receiving TAC. In addition, clinical data on the maintenance of remission with AZA after TAC discontinuation were accessible, facilitating a retrospective comparative study. Second, the sample size was small. Nevertheless, this study was a pilot study, thus necessitating a prospective, randomized, multicenter study in the future, depending on the observed results.

## 5. Conclusions

Switching to ADA as therapy for maintaining remission in patients with refractory UC who achieved clinical remission with TAC therapy was effective in preventing re-lapse. Further investigation of this treatment strategy in a prospective multicenter study with a larger sample size is warranted.

## Figures and Tables

**Table 1:** Demographics and Clinical Characteristics at the Start of TAC.

Number of patients, *n*		17
Male/Female, *n*		10/7
Age, year, median (IQR)		48 (41–64)
Duration of disease, year, median (IQR)		5 (2–15)
UC location; Left side/Extensive, *n*		5/12
Concomitant medications		
Aminosalicylates, *n* (%)		15 (88.2)
Immunomodulator, *n* (%)		5 (29.4)
Corticosteroids, *n* (%)		8 (47.1)
Probiotics, *n* (%)		11 (68.8)
	At the start of TAC	At the start of ADA
Lichtiger index, median (IQR)	13 (10–14)	1 (1–3)
WBC, /μL, median (IQR)	7150 (5700–12,430)	4760 (4160–6070)
Hb, g/dL, median (IQR)	13.2 (10.5–13.9)	12.2 (11.1–14.1)
Albumin, g/dL, median (IQR)	3.4 (2.5–3.7)	4.2 (3.8–4.5)
CRP, mg/L, median (IQR)	1.18 (0.14–10.66)	0.07 (0.03–0.14)
Plt × 10^3^μL, median (IQR)	33.1 (25–42.8)	26.6 (22.4–29.9)

TAC = tacrolimus; UC = ulcerative colitis; WBC = white blood cell; Hb = hemoglobin; CRP = C-reactive protein; Plt = Platelet; IQR = interquartile range; ADA = adalimumab.

**Table 2:** Demographics and Clinical Characteristics at the Start of TAC.

	Total	AZA Group	ADA + AZA Group	*p* Value
Number of patients, *n*	42	26	16	
Male/Female, *n*	25/17	15/11	10/6	0.76
Age, year, median (IQR)	45 (35.5–58.8)	43 (34.3–54.8)	46.5 (40.5–60.25)	0.37
Duration of disease, year, median (IQR)	5 (1–12.75)	4 (1–10)	7 (1.75–17)	0.34
UC location; Left side/Extensive, *n*	7/35	3/23	4/12	0.26
Concomitant medications				
Aminosalicylates, *n* (%)	37 (88.1)	22 (84.6)	15 (93.8)	0.37
Immunomodulator, *n* (%)	10 (23.8)	4 (11.5)	6 (37.5)	0.10
Corticosteroids, *n* (%)	21 (50.0)	13 (50.0)	8 (50.0)	0.69
Probiotics, *n* (%)	28 (66.7)	17 (65.4)	11 (68.8)	0.83
Previous medications before tacrolimus, *n* (%)				
anti-TNF agents; IFX	1 (2.4)	1 (3.8)	0 (0)	0.43
Past history of treatment failure with biologics, *n* (%)				
anti-TNF agents; IFX	1 (2.4)	1 (3.8)	0 (0)	0.43
AZA starting dose	50 (40–50)	50 (40–50)	50 (43.75–50)	1.00
Maximum AZA dose	50 (50–75)	50 (50–100)	62.5 (50–75)	0.86
Lichtiger index, median (IQR)	13 (11–14)	13 (11–15.5)	13 (10–14)	0.54
WBC, /μL, median (IQR)	7690 (6050–10,595)	8315 (6220–10,448)	6660 (5607.5–12,677.5)	0.84
Hb, g/dL, median (IQR)	12.35 (10.83–13.68)	12.05 (10.83–13.58)	13.3 (11.175–13.9)	0.41
Albumin, g/dL, median (IQR)	3.4 (2.6–3.8)	3.2 (2.8–3.8)	3.5 (2.5–3.8)	0.69
CRP, mg/L, median (IQR)	1.25 (0.43–9.015)	1.52 (0.5325–6.24)	0.895 (0.1375–10.023)	0.41
Plt × 10^3^μL median (IQR)	34.7 (28.1–42.7)	34.8 (28.8–41.5)	34.55 (26.875–46.175)	0.65

TAC = tacrolimus; ADA = adalimumab; AZA = azathioprine; IFX = infliximab; TNF = tumor necrosis factor; UC = ulcerative colitis; WBC = white blood cell; Hb = hemoglobin; CRP = C-reactive protein; Plt = platelet; IQR = interquartile range.

**Table 3:** Demographics and Clinical Characteristics at the Start of TAC.

	Remission Group	Relapse Group	*p* Value
Number of patients, *n*	15	27	
Male/Female, *n*	7/8	18/9	0.21
Age, year, median (IQR)	54 (34.5–61.5)	42 (38–53.5)	0.44
Duration of disease, year, median (IQR)	5 (1–11)	5 (1.5–13.5)	0.85
UC location; Left side/Extensive, *n*	1/14	6/21	0.39
Medications for UC taken at baseline			
Aminosalicylates, *n* (%)	14 (93.3)	23 (85.2)	0.43
Immunomodulator, *n* (%)	3 (20)	5 (18.5)	1.00
Corticosteroids, *n* (%)	6 (40.0)	15 (55.6)	0.33
Probiotics, *n* (%)	10 (66.7)	18 (66.7)	0.59
Previous medications before tacrolimus, *n* (%)			
anti-TNF agents; IFX	1(6.7)	0 (0)	0.36
Past history of treatment failure with biologics, *n* (%)			
anti-TNF agents; IFX	1 (6.7)	0 (0)	0.36
Lichtiger index, median (IQR)	13 (10–14)	13 (11–16)	0.37
WBC, /μL, median (IQR)	8830 (6050–12,365)	7410 (6050–10,315)	0.36
Hb, g/dL, median (IQR)	13.2 (11.1–13.9)	12.1 (10.55–13.6)	0.49
Albumin, g/dL, median (IQR)	3.2 (2.5–3.65)	3.6 (2.8-3.8)	0.49
CRP, mg/L, median (IQR)	2.76 (0.46–13.36)	0.82 (0.38–4.245)	0.24
Plt × 10^3^μL, median (IQR)	41.5 (32.7–49.4)	32.3 (23.6–40.7)	0.15
Maintenance therapy after discontinuation of TAC			
AZA, *n* (%)	15 (100)	27 (100)	-
ADA + AZA, *n* (%)	9 (56.3)	7 (43.8)	0.03

TAC = tacrolimus; UC = ulcerative colitis; WBC = white blood cell; Hb = hemoglobin; CRP = C-reactive protein; Plt = Platelet; IQR = interquartile range; TNF = tumor necrosis factor; IFX = infliximab; ADA = adalimumab; AZA = azathioprine.

**Table 4:** Univariate analysis of predictive factors associated with maintenance of clinical remission after TAC discontinuation.

Predictive Factor	Univariate Odds Ratio (95%CI)	*p* Value
ADA + AZA administration	4.3 (1.1–16.4)	0.034

## Data Availability

The data are not publicly available because there is no appropriate site for uploading them at present. The data underlying this article will be shared upon reasonable request to the corresponding author.
